# Inhibition of the hERG Potassium Channel by a Methanesulphonate-Free E-4031 Analogue

**DOI:** 10.3390/ph16091204

**Published:** 2023-08-24

**Authors:** Matthew V. Helliwell, Yihong Zhang, Aziza El Harchi, Christopher E. Dempsey, Jules C. Hancox

**Affiliations:** 1 School of Biochemistry, Biomedical Sciences Building, University Walk, University of Bristol, Bristol BS8 1TD, UK; mhelliwell1@yahoo.com (M.V.H.); c.dempsey@bristol.ac.uk (C.E.D.); 2 School of Physiology, Pharmacology and Neuroscience, Biomedical Sciences Building, University Walk, University of Bristol, Bristol BS8 1TD, UK; yh.zhang@bristol.ac.uk (Y.Z.); aziza.elharchi@bristol.ac.uk (A.E.H.)

**Keywords:** E-4031, E-4031-17, heart, hERG, long QT, methanesulphonanilide, mutagenesis, molecular docking, potassium channel

## Abstract

*hERG* (*human Ether-à-go-go Related Gene*)-encoded potassium channels underlie the cardiac rapid delayed rectifier (I_Kr_) potassium current, which is a major target for antiarrhythmic agents and diverse non-cardiac drugs linked to the drug-induced form of long QT syndrome. E-4031 is a high potency hERG channel inhibitor from the methanesulphonanilide drug family. This study utilized a methanesulphonate-lacking E-4031 analogue, “E-4031-17”, to evaluate the role of the methanesulphonamide group in E-4031 inhibition of hERG. Whole-cell patch-clamp measurements of the hERG current (I_hERG_) were made at physiological temperature from HEK 293 cells expressing wild-type (WT) and mutant hERG constructs. For E-4031, WT I_hERG_ was inhibited by a half-maximal inhibitory concentration (IC_50_) of 15.8 nM, whilst the comparable value for E-4031-17 was 40.3 nM. Both compounds exhibited voltage- and time-dependent inhibition, but they differed in their response to successive applications of a long (10 s) depolarisation protocol, consistent with greater dissociation of E-4031-17 than the parent compound between applied commands. Voltage-dependent inactivation was left-ward voltage shifted for E-4031 but not for E-4031-17; however, inhibition by both compounds was strongly reduced by attenuated-inactivation mutations. Mutations of S6 and S5 aromatic residues (F656V, Y652A, F557L) greatly attenuated actions of both drugs. The S624A mutation also reduced I_hERG_ inhibition by both molecules. Overall, these results demonstrate that the lack of a methanesulphonate in E-4031-17 is not an impediment to high potency inhibition of I_hERG_.

## 1. Introduction

Potassium channels encoded by *hERG* (*human Ether-à-go-go Related Gene*; alternative nomenclature *KCNH2*) play an important role in cardiac action potential (AP) repolarisation [1] and, thereby, also in setting the duration of the QT interval (the time from the start of the Q wave to the end of the T wave on the electrocardiogram). The hERG protein forms tetrameric channels that underlie the cardiac rapid delayed rectifier (I_Kr_) potassium current [2,3]. Loss-of-function *hERG* mutations give rise to the LQT2 form of long QT syndrome [4,5] and gain-of-function *hERG* mutations are responsible for the SQT1 form of short QT syndrome [6,7,8,9]. hERG is the principal repolarisation-modifying target of Class Ia and Class III antiarrhythmic drugs [4,10,11]. However, it is also an unwanted target for numerous chemically and structurally diverse non-cardiac drugs that are linked to the drug-induced form of long QT syndrome (diLQTS) and to the potentially fatal arrhythmia *torsades de pointes* (TdP) [4,12,13]. The link between pharmacological inhibition of hERG channels and diLQTS is sufficiently strong that all novel pharmaceuticals must undergo testing for I_Kr_/hERG channel inhibition [4,12,14].

The archetypal selective inhibitors of I_Kr_/hERG channels come from the methanesulphonanilide family, which includes the antiarrhythmic drugs sotalol and dofetilide, and the experimental agents MK-499 and E-4031 [10]. E-4031 is a potent inhibitor of I_Kr_/hERG and its use was central to the study that first separated pharmacologically distinct cardiac rapid and slow delayed rectifier K^+^ currents [15]. E-4031, dofetilide, and MK-499 have all been employed in experimental studies that identified key molecular determinants on the hERG channel for high-affinity pharmacological inhibition [16,17,18]. The hERG-blocking effects of methanesulphonanilides are highly sensitive to S6 domain mutations (notably to aromatic residues Y652 and F656) and to mutations of residues at the base of the pore helix [16,17,18]. More recent work has identified an S5 aromatic residue F557 as an additional blocking determinant for dofetilide and other high-affinity hERG inhibitors [19,20,21], though its role in E-4031 inhibition has not yet been established.

**Figure 1:**
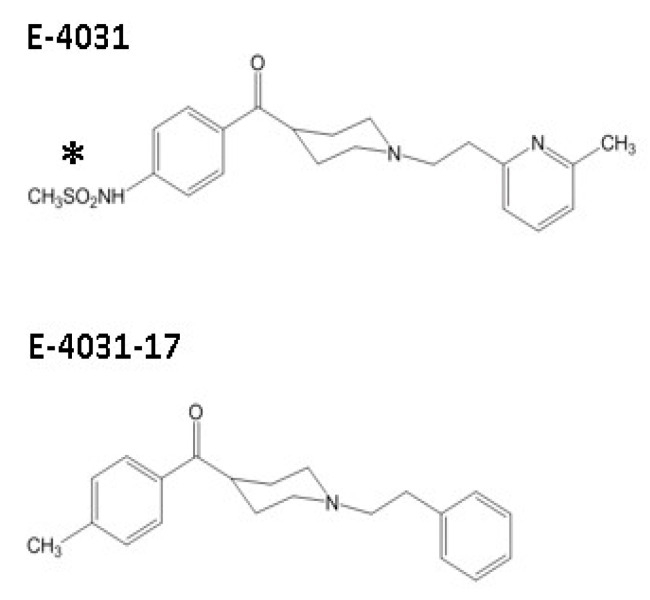
Structures of E-4031 and E-4031-17. Upper panel shows E-4031, asterisk highlights the methanesulphonate group. Lower panel shows E-4031-17.

Although the mutation of the two polar pore helix residues on hERG (T623 and S624) significantly affects methanesulphonanilide block, their importance to hERG channel inhibition appears to differ between drugs [16,18,22,23]. The results of comparative studies of ibutilide and clofilium (structurally related compounds that differ in the *para*-substituents on their respective phenyl rings: ibutilide has a methanesulphonate group whilst clofilium has a chlorine) and of a series of analogues strongly suggested that T623 and S624 directly interact with *para*-substituents of drugs, and that observed differences in block by ibutilide and clofilium are due to the specific properties of the *para*-substituent [24,25]. However, results from experiments on E-4031 analogues from which polar substituents have been removed have shown enhanced binding affinity for hERG, suggesting that polar *para*-substituents are not required [26]. One E-4031 analogue, “E-4031-17”, lacks a methanesulphonate group (see Figure 1), but was reported to have an apparent higher binding affinity to hERG than E-4031 itself in radioligand binding evaluation [26]. This analogue provides a means to investigate the role of the methanesulphonamide group in high-affinity hERG channel inhibition by E-4031 and has not hitherto been subject to systematic electrophysiological evaluation. Consequently, the aim of this study was to compare E-4031 and E-4031-17 inhibition of wild-type (WT) hERG channel current (I_hERG_) and of I_hERG_ carried by channels with mutations to key binding residues [27].

## 2. Results

### 2.1. Concentration Dependence of I_hERG_ Inhibition by E-4031 and Its Analogue E-4031-17

**Figure 2:**
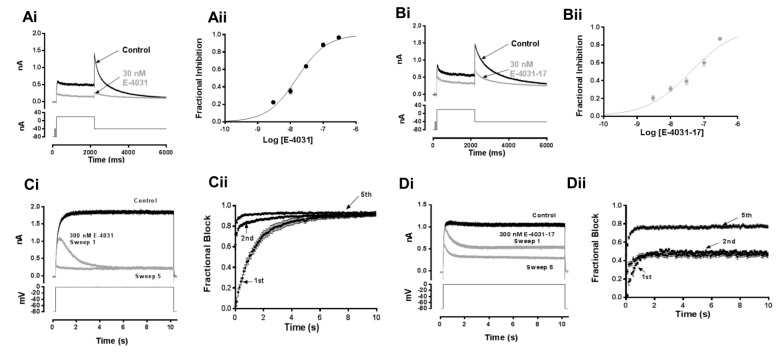
Concentration and time-dependence of I_hERG_ inhibition by E-4031 and E-4031-17. (**Ai**) Representative traces of WT I_hERG_ using the voltage protocol illustrated (lower panel) in Control (black) and after 10 min of superfusion with 30 nM E-4031 (grey). Protocol was applied with a start-to-start interval of 12 s. Concentration-response relationships for fractional inhibition of WT I_hERG_ tails at −40 mV by E-4031. (n ≥ 5 for each point) to give IC_50_ and n_H_ values reported in the Results section (**Aii**). (**B**) Representative traces of WT I_hERG_ in Control (black) and after 10 min of superfusion with 30 nM E-4031-17 (grey). Protocol shown in lower panel (**Bi**). Concentration-response relationships for fractional inhibition of WT I_hERG_ tails at −40 mV by E-4031-17 (n = 5–8 for each point), to give IC_50_ and n_H_ values reported in the Results section (**Bii**). (**C**) Representative I_hERG_ traces elicited by a sustained depolarisation from −80 to 0 mV for 10 s are shown in (**Ci**) in Control and immediately following equilibration in 300 nM E-4031. Four more runs of the protocol were applied in the presence of the drug; only sweeps 1 and 5 are shown for clarity. (**Cii**) Mean (±S.E.M.) data from experiment described in (**Ci**), showing fractional block calculated at 100 ms intervals plotted against the time that the membrane potential was depolarised (n = 6). Mean fractional block data from the first (1st) sweep in the presence of E-4031 were fitted with a mono-exponential association equation to produce a rate constant of *k* = 0.75 ± 0.02 s^−1^ and a time constant of *τ* ≈ 1.3 s. (**D**) Comparable data to ‘C’ for E-4031-17. (**Di**) shows representative I_hERG_ traces in Control and for first, second, and fifth applications of the protocol following equilibration with 300 nM E-4031-17. (**Dii**) shows mean (±S.E.M.) data from experiment described in (**Di**), showing fractional block calculated at 100 ms intervals plotted against the time that the membrane potential was depolarised (n = 6). Data in E-4031-17 were fitted with a mono-exponential association equation to produce a rate constant of *k* = 2.06 ± 0.07 s^−1^ and a time constant of *τ* ≈ 0.49 s.

The inhibition of I_hERG_ by E-4031 and by the E-4031-17 analogue was interrogated using repeated application of a voltage protocol comprising a 2 s depolarisation from −80 mV to +20 mV followed by repolarisation to −40 mV. The amplitude of the I_hERG_ tail elicited on repolarisation was measured relative to the current evoked by a short (50 ms) pre-pulse from −80 to −40 mV, similar to previous pharmacological studies from our laboratory (e.g., [28,29]). Figure 2Ai shows exemplar currents elicited by this protocol in control (Tyrode’s solution) and following 10 min of application of 30 nM E-4031. I_hERG_ during the +20 mV depolarisation step and tail current on repolarisation to −40 mV were markedly inhibited by this E-4031 concentration. Similar experiments were performed with a total of five E-4031 concentrations (3, 10, 30, 100, and 300 nM). For each concentration, fractional inhibition of I_hERG_ was determined (Equation (1), Methods) and mean data were then plotted (Figure 2Aii) and fitted with Equation (2) (Methods) to produce a concentration-response relation. This yielded a half maximal inhibitory concentration (IC_50_) value of 15.8 nM (CI: 13.7–18.2) and a Hill co-efficient (n_H_) of 0.96 (CI: 0.84–1.09) (n ≥ 5 for each concentration). This IC_50_ value is similar to prior IC_50_ values for E-4031 obtained at 35–37 °C (7.7–16.0 nM) [30,31]. Figure 2Bi,Bii show comparable data for E-4031-17. Figure 2Bi shows that 30 nM E4031-17 inhibited I_hERG_ throughout the voltage protocol, but to a smaller extent than observed in Figure 2Ai,Aii for the parent molecule. Figure 2Bii shows mean data for the same five concentrations as used for E-4031. The derived IC_50_ value for E-4031-17 was 40.3 nM (CI: 30.8–52.7), with an n_H_ of 0.66 (CI: 0.53–1.09; n = 5–8 for each concentration). Both the IC_50_ and n_H_ values differed significantly (*p* < 0.0001 and *p* < 0.001, respectively; unpaired *t*-test with Welch’s correction) between E-4031 and E-4031-17.

### 2.2. I_hERG_ Inhibition by E-4031 and E-4031-17 during a Sustained Depolarisation

The time-dependence of development of I_hERG_ on channel gating can be evaluated using the application of a protocol comprising a sustained membrane depolarisation (e.g., [28,32]). A 10 s duration depolarisation (lower panels of Figure 2Ci,Di) was applied in control solution before continuous superfusion with 300 nM of E-4031 (Figure 2Ci,Cii) or E-4031-17 (Figure 2Di,Dii), with the membrane potential held at −80 mV. Following drug equilibration, the protocol was then reapplied five times. Figure 2Ci,Di, respectively, show representative traces for E-4031 and E-4031-17. During drug equilibration at −80 mV, hERG channels are anticipated to be closed; consequently, the first application of the protocol enables drug association to channels moving from closed to gated states during the protocol. For both compounds, little or no block was evident immediately on membrane potential depolarisation (zero fractional inhibition at time 0 in Figure 2Cii,Dii for the first protocol application). Inhibition then progressively developed during the sustained depolarisation. Mean fractional block data from the first sweep in the presence of the two compounds were fitted with a mono-exponential association equation to produce a rate constant (k) = 0.75 ± 0.02 s^−^^1^ (a time constant of τ ≈ 1.3 s) for E-4031 and a k of 2.06 ± 0.07 s^−^^1^ (a time constant of τ ≈ 0.49 s) for E-4031-17. For E-4031, the second protocol application resulted in a much greater extent of initial block, with a further small amount of block developing with time; there was a further small increase in initial block for the fifth application of the protocol. Notably, for E-4031, the fractional inhibition of I_hERG_ was similar by 10 s for the first, second, and fifth applications of the protocol (Figure 2Cii). By contrast, for E-4031-17, fractional block following initial depolarisation was greater during the second pulse application, with block converging with that occurring during the first pulse application by the end of the voltage command (Figure 2Dii). Greater initial and end pulse inhibition was evident for the fifth application of the protocol (Figure 2Dii). These data show that I_hERG_ block by both E-4031 and E-4031-17 was contingent upon channel gating; the observed differences in Figure 2Cii,Dii on repeated application of the protocol are suggestive that there was greater relief of block between successive protocol applications for E-4031-17 than for the parent compound.

### 2.3. Effects of E-4031 and E-4031-17 on I_hERG_ Elicited at Different Test Voltages

**Figure 3:**
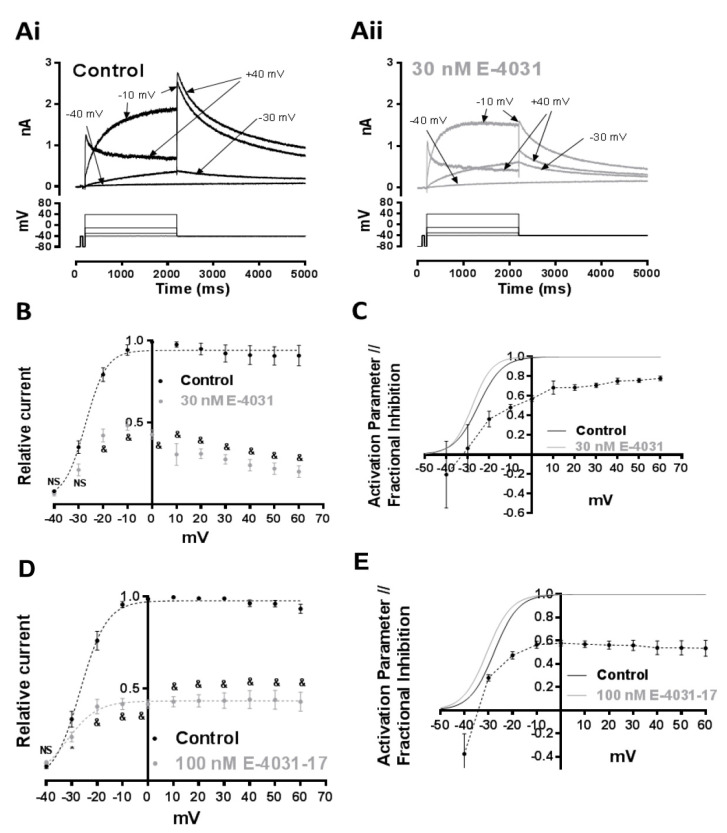
Effects of E-4031 and E-4031-17 at different test voltages (**A**) Representative WT I_hERG_ traces elicited by the I–V protocol illustrated (lower panel) in Control (**Ai**) and in the presence of 30 nM E-4031 (**Aii**). The membrane potential was depolarised from a holding potential of −80 mV to a range of different test potentials (−40 mV to +60 mV) in 10 mV increments. This was followed by repolarisation to −40 mV, which elicited resurgent I_hERG_ tails used to assess current-voltage (I-V) relationships (start-to-start interval = 12 s). (**B**) Normalised I–V relationships for hERG I_tails_ in Control (black) and in the presence of 30 nM E-4031 (grey). Peak tail currents before and after application of 30 nM E-4031 were normalised to the peak tail current amplitude in Control (n = 5, ‘&’ signifies *p* < 0.05; 2 Way ANOVA with Bonferroni post hoc test). Experimental points were fitted with a Boltzmann function (Equation (3), Methods). Control V_0.5_ = −25.0 ± 1.6 mV, k = 4.3 ± 0.8, and 30 nM E-4031 V_0.5_ = −27.8 ± 2.0 mV, k = 4.6 ± 2.1. (n = 5). (**C**) Voltage-dependence of I_hERG_ block by E-4031 (black dotted line), with superimposed voltage-dependent activation relationships for I_hERG_ in Control (black continuous line) and after application of 30 nM E-4031 (grey line); n = 5. (**D**) Comparable mean ±S.E.M. data for E-4031-17 to that shown in (**B**) for E-4031. The concentration of E-4031-17 used (100 nM) was selected from the concentration-response relations in Figure 2 to be sufficient to produce > 50%, but incomplete inhibition of I_hERG_ Control (n = 5, & signifies *p* < 0.05; 2-Way ANOVA with Bonferroni post hoc test). Experimental points were fitted with Equation (3), Methods. Control V_0.5_ = −26.7 ± 0.4 mV, k = 5.2 ± 0.4, and 100 nM E-4031-17 V_0.5_ = −32.0 ± 1.6 mV, k = 6.0 ± 1.5. (n = 5). (**E**) Voltage-dependence of I_hERG_ block by E4031-17 (black dotted line) and voltage-dependent activation relationships for I_hERG_ in Control (black continuous line) and after application of 30 nM E-4031-17 (grey line); n = 5.

Further experiments were conducted in which I_hERG_ was elicited by 2 s duration voltage commands to a range of test potentials between −40 and + 60 mV; the extent of inhibition of the I_hERG_ tails at −40 mV following the different commands was then determined (cf [29]). Figure 3Ai,Aii show representative currents elicited by this protocol (at the selected potentials shown) in control solution and in the presence of 30 nM E-4031. In the examples shown, there was no reduction in I_hERG_ evident for a test potential of −30 mV (with modest increases in end pulse and tail current amplitude visible), but with marked reductions in currents for the −10 and +40 mV test potentials. I_hERG_ tail amplitudes in control and in the presence of 30 nM E-4031 were normalised to the maximal current recorded in control during the protocol for each cell and plotted against the membrane potential of the depolarising steps. Figure 3B shows the resulting normalised current voltage (I–V) relations. These data were fitted with a Boltzmann equation (Equation (3); Methods) to obtain activation V_0.5_ values of −25.0 ± 1.6 mV (control) and −27.8 ± 2.0 mV (E-4031) with respective slope (k) factors of 4.3 ± 0.8 and 4.6 ± 2.1 (n = 5; *p* > 0.05 vs. control for both). It should be noted that there was a greater effect of E-4031 at positive potentials; therefore, the Boltzmann sigmoidal slope could only be fitted between −40 mV and −10 mV in E-4031 and for clarity of display is omitted from Figure 3B. Differences between I_hERG_ tail amplitude in control and E-4031 were significant at all potentials except −40 and −30 mV. As the test potentials became more positive, the I–V relations diverged (*p* < 0.001, 2-WAY ANOVA with Bonferroni post hoc test), consistent with voltage-dependent inhibition. In Figure 3C, fractional block of tail currents following each test potential is plotted against test potential. The V_0.5_ and k values obtained from fitting the I–V data in Figure 3B were used to simulate activation variables at 2 mV intervals and the resulting activation relations in control and E-4031 are also plotted in Figure 3C [29]. The Figure shows that the steepest range of voltage-dependence of I_hERG_ inhibition coincided with the rising phase of the superimposed activation relations, as expected for a drug with gating (activation)-dependent inhibition. Similar experiments were conducted using 100 nM E-4031-17, with mean data (n = 5) plotted in Figure 3D,E. The normalised I–V relations plotted in Figure 3D show a significant reduction in I_hERG_ tail amplitude occurred at all test potentials positive to −30 mV. Boltzmann fits to the I–V relations gave a control V_0.5_ value of −26.7 ± 0.4 mV (k of 5.2 ± 0.4) and in E-4031-17 a V_0.5_ of −32.0 ± 1.6 mV and k of 6.0 ± 1.5. The negative shift in activation V_0.5_ by 100 nM E-4031-17 was statistically significant (*p* < 0.0001). The plots in Figure 3E show that the steepest range of voltage-dependent block of I_hERG_ by E-4031-17 corresponded to the rising phase of the activation relations for I_hERG_, with block levelling out at positive voltages. The key finding from these experiments is that E-4031-17, like its parent compound, is an activation-dependent hERG blocker [33].

### 2.4. I_hERG_ Inactivation and the Inhibitory Actions of E-4031 and E-4031-17

An intact inactivation process has been shown to be important for high-affinity hERG channel block, including by methanesulphonanilides, either through increasing drug binding or by facilitating the orientation of the S6 aromatic residues to which drugs bind [34,35,36]. Non-inactivating eag channels that are insensitive to E-4031 have been shown to gain both inactivation sensitivity to the drug when chimeric channels incorporating a polypeptide comprising pore and S6 domain hERG residues were made [37] and inactivation attenuating mutations greatly reduce the inhibitory potency of E-4031 on I_hERG_ (e.g., [31]). 

**Figure 4:**
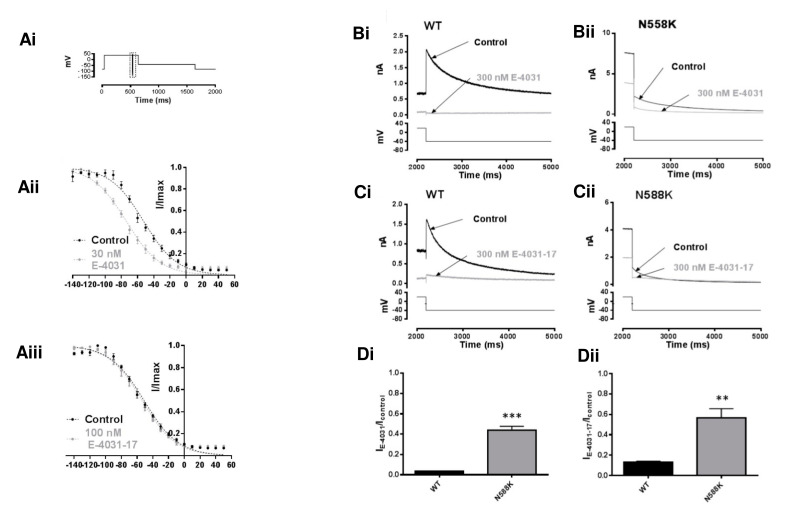
I_hERG_ inactivation and effects of E-4031 and E-4031-17 (**A**) Voltage protocol used to evaluate WT I_hERG_ inactivation is shown in (**Ai**). Membrane potential was depolarised from a holding potential of −80 mV to +40 mV for 500 ms to activate and inactivate I_hERG_. This was followed by a very brief (2 ms) repolarising pulse to a range of potentials (−140 to +50 mV in 10 mV increments for each pulse). This phase enabled I_hERG_ rapidly to recover from inactivation; the extent of recovery was dependent on the repolarising test potential. The membrane potential was then stepped to +40 mV for 100 ms to assess I_hERG_ availability. (**Aii**) shows plots describing voltage-dependence of I_hERG_ inactivation in Control (black circles, black dashed line) and in the presence of 30 nM E-4031 (grey circles, grey dashed line). Data were corrected for deactivation, then fitted with Equation (4) Methods. *V*_0.5_ = −52.4 ± 1.1 mV, *k* = 20.7 ± 0.9 in Control and *V*_0.5_ = −73.7 ± 0.9 mV, *k* = 20.9 ± 0.8 in the presence of 30 nM E-4031 (*p* < 0.05, paired *t*-test, n = 6). (**Aiii**) Voltage-dependence of I_hERG_ inactivation in Control (black circles, black dashed line) and in the presence of 100 nM E-4031-17 (grey circles, grey dashed line). Data were corrected for deactivation, then fit with a Boltzmann equation. *V*_0.5_ = of −51.1 ± 0.8 mV, *k* = 21.6 ± 0.7 in Control and *V*_0.5_ = −53.4 ± 0.9 mV, *k* = 21.2 ± 0.8 in the presence of 100 nM E4031-17 (NS; *p* < 0.05, paired *t*-test, n = 6). (**B**) Effects of N588K mutation on actions of E-4031 and E-4031-17. Representative WT (**Bi**) and N588K (**Bii**) I_hERG_ traces elicited on repolarisation to −40 mV from +20 mV in Control (black) and after application of 300 nM E-4031 (grey). (**C**) Representative WT (**Ci**) and N558K (**Cii**) I_hERG_ traces similar to (**B**) but with 300 nM E-4031-17. (**D**) Bar charts showing means ±S.E.M. normalised I_tails_ at −40 mV after 8 min in Control (black) and in the presence of 300 nM E-4031 (**Di**) or 300 nM E-4031-17 (**Dii**). Values are expressed as I_E-4031_/I_Control_ or I_E-4031-17_/I_Control_ and represent the relative tail current remaining after 8 min of superfusion with drug and after rundown correction. ‘**’ and ‘***’ represent *p* ˂ 0.005 and *p* ˂ 0.0005, respectively; Unpaired *t*-test, n ≥ 5.

Figure 4A shows effects of both compounds on WT I_hERG_ inactivation. The protocol used is shown in Figure 4Ai and is similar to that used in prior studies from our laboratory [20,29]. For this protocol, membrane potential was depolarised from a holding potential of −80 mV to +40 mV for 500 ms to allow the majority of the population of hERG channels to both activate and inactivate. This preceded a very brief (2 ms) repolarising pulse to a range of potentials (−140 to +50 mV in 10 mV increments for each pulse). This repolarisation phase causes hERG channels to rapidly recover from inactivation; the extent of recovery is dependent on the depolarising test potential. The membrane potential was then stepped to +40 mV to assess I_hERG_ availability. The amplitude of the resurgent current elicited by this step reflects the proportion of hERG channels that recovered from inactivation during the brief repolarisation phase. This protocol was applied in the absence and then presence of each of E-4031 or E-4031-17. Current values were then normalised to the maximal peak amplitude and plotted against the voltage of the preceding 2 ms step. The resulting plots were fitted with Equation (4) (Methods) in order to derive voltage-dependent inactivation parameters of I_hERG_. Figure 4Aii shows the effect of 30 nM E-4031. Under control conditions, the V_0.5_ of inactivation was −52.4 ± 1.1 mV (with a k of 20.7 ± 0.9 mV), whereas in the presence of 30 nM E-4031, the V_0.5_ became −73.7 ± 0.9 mV (*p* < 0.05 vs. control, paired *t*-test, n = 6), with a slope factor that was essentially unchanged (k = 20.9 ± 0.8 mV). Comparable data for E-4031-17 (100 nM) are shown in Figure 4Aiii. The inactivation V_0.5_ from these measurements was −51.1 ± 0.8 mV (k = 21.6 ± 0.7) in control and −53.4 ± 0.9 mV (k = 21.2 ± 0.8) in E-4031-17 (*p* > 0.05, paired *t*-test, n = 5). Thus, voltage-dependence of I_hERG_ inactivation was left-ward voltage shifted for E-4031, but not E-4031-17.

I_hERG_ inhibition by E-4031 has previously been shown to be highly sensitive to attenuation of inactivation with a marked decrease in inhibitory potency seen for current carried by the attenuated-inactivation N588K hERG mutant [31]. This residue is located in the external S5-Pore linker region, remote from the methanesulphonanilide binding site on the channel; the N588K mutation positively shifts I_hERG_ inactivation by ~+60 to +90 mV [38,39]. Figure 4B–D compare the effects of the N588K mutation on I_hERG_ inhibition by E-4031 and E-4031-17. Figure 4B,C show representative I_hERG_ tails carried by WT (Bi,Ci) and N588K (Bii,Cii) channels elicited at −40 mV by the ‘standard protocol’ (lower panel). Figure 4Di,Dii contain bar graphs showing the mean values of normalised I_tails_ in control and after application of 300 nM E-4031 (4Di) and E-4031-17 (4Dii), respectively. While 300 nM E-4031 profoundly reduced WT hERG I_tail_ amplitude to 3.4 ± 0.7% of the control amplitude (n = 6), N588K hERG I_tail_ was reduced to 43.9 ± 7.6% of control (n = 5; *p* ˂ 0.0005). Thus, the N588K mutation significantly reduced I_hERG_ inhibition by E-4031 (Figure 4Di). 300 nM E-4031-17 reduced WT hERG I_tail_ amplitude to 12.9 ± 2.8% of control (n = 7), whereas N588K hERG I_tail_ was reduced to 43.2 ± 2.0% (n = 6; *p* ˂ 0.005, unpaired *t*-test; Figure 4Dii). Thus, the inhibitory actions of both compounds were reduced for the attenuated-inactivation mutant. For completeness, further experiments (data not shown) were performed using a second mutation (S620T; to a residue located in the pore helix), which completely abolishes I_hERG_ inactivation [40]. 300 nM E-4031 reduced the S620T hERG I_tail_ amplitude to 64.3 ± 5.0% of control (n = 6) compared to 3.4 ± 0.7% of control for the WT channel (n = 6; *p* ˂ 0.0001). 300 nM E-4031-17 reduced the S620T hERG I_tail_ amplitude to 69.3 ± 4.0% of control (n = 5) compared to 12.9 ± 2.8% of control for the WT channel (n = 7; *p* ˂ 0.0001). The key observation from these experiments is that I_hERG_ block by E-4031-17 resembles that of the parent compound in its dependence on an intact inactivation process.

### 2.5. Effects of Mutations at the Base of the Pore Helix on E-4031 and E-4031-17

**Figure 5:**
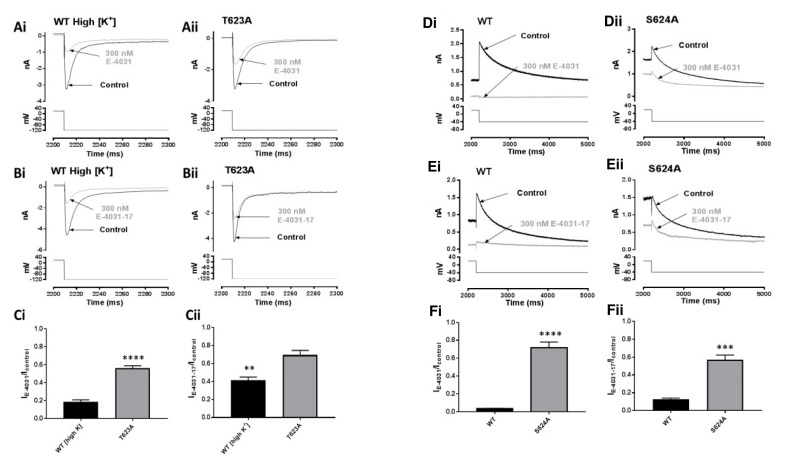
Effects of mutations at the base of the pore helix on actions of E-4031 and E-4031-17. (**A**) Representative WT (**Ai**) and T623A (**Aii**) hERG inward I_tails_ elicited at −120 mV in 94 mM [K^+^]_e_ in Control and in the presence of 300 nM E-4031 (**Aii**). The traces and voltage protocol (lower panels) are expanded at the end of the depolarising step to +20 mV and the beginning of the hyperpolarising step to −120 mV for clarity. (**B**) Representative WT (**Bi**) and T623A (**Bii**) I_hERG_ traces similar to (**A**) but with 300 nM E-4031-17. (**C**) Bar charts showing means ±S.E.M. normalised inward I_tails_ at −120 mV after 8 min in Control (black) or in the presence of 300 nM E-4031 (**Ci**) or 300 nM E-4031-17 (**Cii**). Values are expressed as I_E-4031_/I_Control_ and I_E-4031-17_/I_Control_, and represent the relative tail current remaining after 8 min of superfusion with drug and after rundown correction. ‘**’ and ‘****’ represent *p* ˂ 0.005 and *p* ˂ 0.0001, respectively; Unpaired *t*-test, n ≥ 5. (**D**) Representative WT **(Di)** and S624A **(Dii)** hERG I_tails_ elicited at −40 mV using the ‘standard protocol’ in Control (**Di**) and in the presence of 300 nM E-4031 (**Dii**). (**E**) Representative WT (**Ei**) and S624A (**Eii**) I_hERG_ traces similar to (**D**) but with 300 nM E-4031-17. (**F**) Bar charts showing means ±S.E.M. normalised I_tails_ at −40 mV after 8 min in Control (black) or in the presence of 300 nM E-4031 (**Fi**) or 300 nM E-4031-17 (**Fii**). Values are expressed as I_E-4031_/I_Control_ or I_E-4031-17_/I_Control_ and represent the relative tail current remaining after 8 min of superfusion with drug and after rundown correction. ‘***’ and ‘****’ represent *p* ˂ 0.0005 and *p* ˂ 0.0001, respectively; Unpaired *t*-test, n ≥ 5.

I_hERG_ inhibition by E-4031 has previously been shown to be sensitive to mutations of the T623 and S624 residues at the base of the pore helix of hERG [18]. Therefore, we evaluated the effects of alanine mutants at these positions on E-4031 and E-4031-17 inhibition of I_hERG_. Measurement of I_hERG_ carried by T623A channels requires high [K^+^]_e_ and recording of inward I_hERG_ [16,29]. Figure 5A,B show representative inward I_hERG_ tails conducted by WT (Figure 5Ai,Bi) and T623A (Figure 5Aii,Bii) in control and after application of 300 nM E-4031 and 300 nM E-4031-17, respectively. The bar graphs in Figure 5Ci,Cii represent pooled mean values of normalised inward I_tails_ in control and after application of 300 nM E-4031 and 300 nM E-4031-17, respectively. E-4031 reduced the inward T623A hERG I_tail_ amplitude to 56.2 ± 6.2% of control amplitude (n = 5) compared to 18.4 ± 4.9% for the WT channel (n = 6; *p* ˂ 0.0001, unpaired *t*-test). 300 nM E-4031-17 reduced the inward T623A hERG I_tail_ amplitude to 69.7 ± 9.9% of control amplitude (n = 5) compared to 41.5 ± 7.1% for the WT channel (n = 7; *p* ˂ 0.005, unpaired *t*-test). These effects, although statistically significant, were comparatively modest compared to those shown in Figure 4 for the N588K mutation. It is also notable that inhibition of inward WT I_hERG_ in high [K^+^]_e_ was greater for E-4031 than for E-4031-17 (Figure 5Ci,Cii; *p* < 0.05 for comparison of fractional block by the two compounds). 

The S624A mutation reduces the hERG blocking potency of compounds that include clofilium, dofetilide, E-4031, ibutilide, vesnarinone, cisapride, and terfenadine [18,41,42]. Figure 5D,E show representative I_hERG_ traces from WT (Di,Ei) and S624A (Dii,Eii) hERG elicited at −40 mV by the ‘standard protocol’ (lower panel) in control and in the presence of 300 nM E-4031 and 300 nM E-4031-17, respectively. The bar graphs in Figure 5Fi,Fii show mean normalised I_tails_ values in control and after application of 300 nM E-4031 and 300 nM E-4031-17. 300 nM E-4031 reduced the S624A hERG I_tail_ amplitude to 71.5 ± 10.7% (n = 7) of its control compared to 3.4 ± 0.7% for WT hERG (n = 5; *p* ˂ 0.0001). 300 nM E-4031-17 reduced the S624A hERG I_tail_ amplitude to 57.2 ± 10.2% (n = 6) compared to 12.9 ± 2.8% for WT hERG (n = 7; *p* = 0.0001).

### 2.6. Effects of Mutation of S6 and S5 Aromatic Residues on Actions of E-4031 and E-4031-17

**Figure 6:**
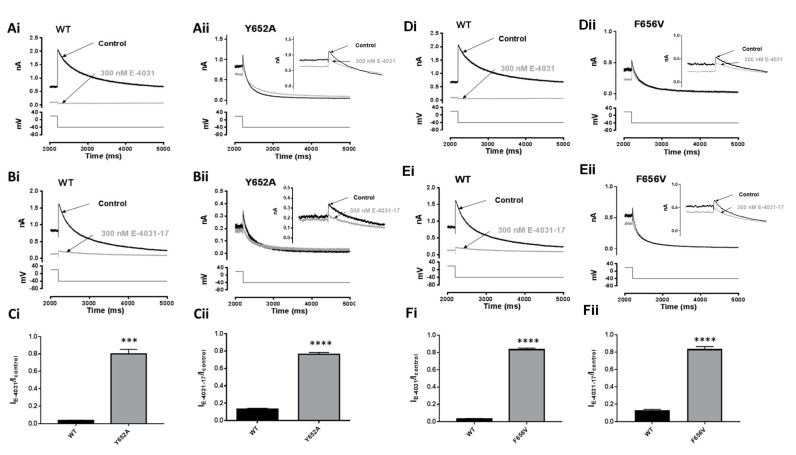
Effects of mutations of S6 aromatic residues on actions of E-4031 and E-4031-17. (**A**) Representative WT (**Ai**) and Y652A (**Aii**) hERG I_tails_ elicited at −40 mV using the ‘standard protocol’ in Control and in the presence of 300 nM E-4031. (**B**) Representative WT (**Bi**) and Y652A (**Bii**) I_hERG_ traces similar to (**A**) but with 300 nM E-4031-17. (**C**) Bar charts showing means ±S.E.M. normalised I_tails_ at −40 mV after 8 min in Control (black) or in the presence of 300 nM E-4031 (**Ci**) or 300 nM E-4031-17 (**Cii**). Values are expressed as I_E-4031_/I_Control_ or I_E-4031-17_/I_Control_, and represent the relative tail current remaining after 8 min of superfusion with drug and after rundown correction. ‘***’ and ‘****’ represent *p* ˂ 0.0005 and *p* ˂ 0.0001, respectively; Unpaired *t*-test, n ≥ 5. (**D**) Representative WT (**Di**) and F656V (**Dii**) hERG I_tails_ elicited at −40 mV using the ‘standard protocol’ in Control and in the presence of 300 nM E-4031. (**E**) Representative WT (**Ei**) and F656V (**Eii**) I_hERG_ traces similar to (**D**) but with 300 nM E-4031-17. (**F**) Bar charts showing means ±S.E.M. normalised I_tails_ at −40 mV after 8 min in Control (black) or in the presence of 300 nM E-4031 (**Fi**) or 300 nM E-4031-17 (**Fii**). Values are expressed as I_E-4031_/I_Control_ or I_E-4031-17_/I_Control_, and represent the relative tail current remaining after 8 min of superfusion with drug and after rundown correction. ‘‘****’ represents *p* ˂ 0.0001; Unpaired *t*-test, n ≥ 5.

Y652 is an S6 polar residue poorly conserved in Kv channels [43] that has been identified as a key determinant for pharmacological blockade of hERG by multiple diverse drugs [4], including E-4031 [18]. Figure 6A,B show representative I_hERG_ tails conducted by WT (Ai, Bi) and Y652A (Aii, Bii) channels elicited at −40 mV (lower panel) in Control and in the presence of 300 nM E-4031 or 300 nM E-4031-17 (grey). The bar graphs in Figure 6Ci,Cii show the mean values of normalised I_tails_ in control and after application of 300 nM E-4031 and 300 nM E-4031-17. 300 nM E-4031 reduced the Y652A hERG I_tail_ amplitude to 79.9 ± 10.6% of control amplitude (n = 5) compared to a corresponding value of 3.4 ± 0.7% for the WT channel (n = 5; *p* ˂ 0.0005). 300 nM E-4031-17 reduced the Y652A hERG I_tail_ amplitude to 76.2 ± 4.3% (n = 5) of control amplitude compared to 12.9 ± 2.8% for the WT channel (n = 7; *p* ˂ 0.0001).

The F656 S6 hydrophobic residue is conserved within the eag channel family [43]; mutations to F656 have been shown strongly to attenuate the inhibition of hERG by most drugs tested, including E-4031 [4,16,17,18]. Figure 6D,E show representative I_hERG_ tail traces for WT (Di,Ei) and F656V (Dii,Eii) channels elicited at −40 mV (protocol in lower panel) and in the presence of 300 nM E-4031 and 300 nM E-4031-17 (grey), respectively. The bar graphs in Figure 6Fi,Fii show mean normalised I_tails_ in control and after application of 300 nM E-4031 and 300 nM E-4031-17. 300 nM E-4031 reduced the F656V hERG I_tail_ amplitude to 83.2 ± 3.6% of control amplitude (n = 5) compared to 3.4 ± 0.7% for WT hERG (n = 5; *p* ˂ 0.0001, unpaired *t*-test). 300 nM E-4031-17 reduced the F656V hERG I_tail_ amplitude to 83.5 ± 5.2% of control (n = 6) compared to 12.9 ± 2.8% for the WT channel (n = 7; *p* ˂ 0.0001).

**Figure 7:**
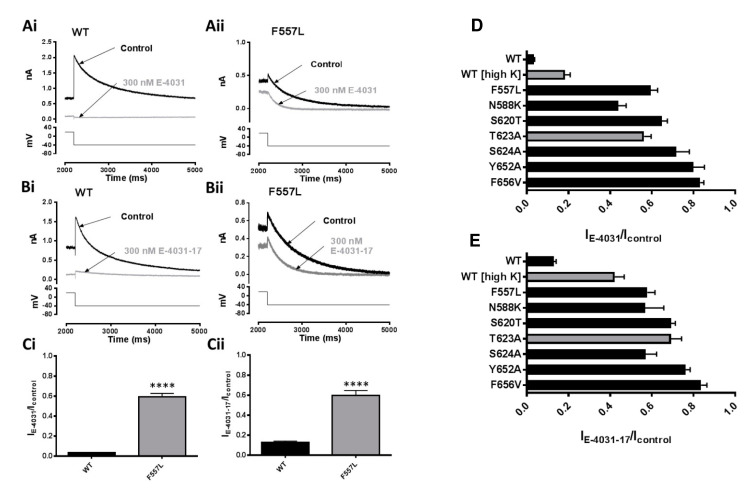
Effect of the S5 F557L mutation on actions of E-4031 and E-4031-17 (**A**) Representative WT (**Ai**) and F557L (**Aii**) hERG I_tails_ elicited at −40 mV using the ‘standard protocol’ in Control and in the presence of 300 nM E-4031. (**B**) Representative WT (**Bi**) and F557L (**Bii**) I_hERG_ traces similar to (**A**) but with 300 nM E-4031-17. (**C**) Bar charts showing means ±S.E.M. normalised I_tails_ at −40 mV after 8 min in Control (black) or in the presence of 300 nM E-4031 (**Ci**) or 300 nM E-4031-17 (**Cii**). Values are expressed as I_E-4031_/I_Control_ or I_E-4031-17_/I_Control_, and represent the relative tail current remaining after 8 min of superfusion with drug and after rundown correction. ‘‘****’ represents *p* ˂ 0.0001; Unpaired *t*-test, n ≥ 5. (**D**) Normalised current (I_E-4031_/I_Control_) measured after steady state block by 300 nM E-4031 (n ≥ 5; error bars represent S.E.M). (**E**) Normalised current (I_E-4031-17_/I_Control_) measured after steady state block by 300 nM E-4031-17 (n ≥ 5; error bars represent S.E.M). Bars in (**D**,**E**) shaded grey indicates currents that were recorded in High [K^+^]_e_ (as shown in Figure 5).

The F557 S5 hydrophobic residue is located in close apposition to Y652 in S6 and has been shown to reduce I_hERG_ inhibition by a number of compounds including dofetilide [19,20,21]. To our knowledge, effects of the mutation of F557 on E-4031 inhibition of I_hERG_ have not yet been reported. We employed the F557L mutation (as used in previous studies [19,20,21]) to investigate the role of this residue in E-4031 and E-4031-17 actions. Figure 7A,B show representative I_hERG_ tails from WT (Ai,Bi) and F557L (Aii,Bii) hERG (protocol in lower panel) in control and in 300 nM E-4031 and 300 nM E-4031-17, respectively. The bar charts in Figure 7Ci,Cii show mean normalised I_tails_ in control and after application of 300 nM E-4031 and 300 nM E-4031-17. E-4031 reduced the F557L hERG I_tail_ amplitude to 59.5 ± 6.6% of control (n = 5) compared to 3.4 ± 0.7% for WT I_hERG_ (n = 5; *p* ˂ 0.0001). 300 nM E-4031-17 reduced the F557L hERG I_tail_ amplitude to 57.9 ± 6.7% of control (n = 6) compared to 12.9 ± 2.8% for the WT channel (n = 7; *p* ˂ 0.0001).

Figure 7D,E summarize the effects of E-4031 and E-4031-17 on WT and mutant I_hERG_, with the size of the horizontal bars denoting the fraction of current remaining in the presence of E-4031. Overall, the results were similar for the two compounds with attenuation of inactivation and mutations of aromatic S5 and S6 residues having large effects on observed inhibition. The level of WT I_hERG_ block by both compounds was reduced for inward I_hERG_ in high [K^+^] compared to outward I_hERG_ with normal [K^+^]_,_ particularly for E-4031-17, with a relatively small further decrease in block by the T623A mutation. 

## 3. Discussion

### 3.1. Contextualizing the Inhibitory Potency of E-4031-17

The potency of E-4031 inhibition of I_hERG_ found in this study is similar to that in prior investigations using mammalian cell line hERG expression [30,31]. Comparable electrophysiological data for E-4031-17 have not hitherto been reported. Results from [^3^H]-astemizole binding experiments suggested increased potency of E-4031-17 compared to the parent compound [26]. However, our electrophysiological comparison showed a modest reduction of I_hERG_ inhibition by E-4031-17 against WT I_hERG_. Astemizole interacts with canonical binding residues in the hERG channel pore [19,44] and a validation study of [^3^H]-astemizole binding potencies found a similar potency rank order for a series of reference compounds to those reported for hERG channel inhibition [45]. Nevertheless, although radioligand binding has advantages in terms of simplicity and compound throughput, electrophysiological evaluation of hERG channel inhibition remains the gold-standard approach, allowing direct evaluation of ionic current [12]. Vilums et al. reported an IC_50_ of 124 nM for I_hERG_ inhibition by E-4031 using an automated patch-clamp and an IC_50_ from radioligand binding of 249 nM [26]. While our I_hERG_ IC_50_ for E-4031-17 of 40.3 nM is in fair agreement with the prior value of 28 nM for this compound from radioligand binding [26], the I_hERG_ IC_50_ value for E-4031 from the automated patch-clamp of hERG-expressing CHO-K1 cells reported by Vilums et al. [26] is nearly eight-fold our manual patch-clamp value of 15.8 nM. By comparison, an independent study of the hERG-expressing HEK 293 cell line used for this study reported an I_hERG_ IC_50_ for E-4031 of 7.7 nM [30], and in experiments on CHO-cells expressing WT hERG, we previously found an IC_50_ for I_hERG_ inhibition of 15.96 nM [31]. A very recent study of WT hERG in HEK 293 cells reported I_hERG_ IC_50_ values for E-4031 of 20.7 nM at room temperature and 12.5 nM at near physiological temperature [46]. The values from these studies are close to the IC_50_ for E-4031 against WT I_hERG_ in the present study. The basis for the substantial difference between such values and the high E-4031 I_hERG_ IC_50_ reported by Vilums et al. [26] is not clear. However, the comparatively low potency of E-4031 observed in the study of Vilums et al. may account for why E-4031-17 was considered to be more potent than the parent compound in that study but was not found to be so in the present one. The relative potencies of I_hERG_ inhibition by E-4031 and E-4031-17 seen here compared to those from radioligand binding [26] underscore the importance of direct, comparative electrophysiological evaluation of compounds. Importantly, the I_hERG_ IC_50_ values for both E-4031 and E-4031-17 seen here are indicative of high potency hERG inhibition. Therefore, a major conclusion from our study is that the lack of a methanesulphonate group in E-4031-17 did not convert high potency to low potency inhibition.

### 3.2. E-4031—Comparison with Prior Experimental Studies

Data from Xenopus oocyte recordings showed that both MK-499 and E-4031 exerted little inhibitory effect on resting hERG channels [33]. Inactivation attenuation was subsequently shown to decrease the affinity of E-4031 for hERG, and I_hERG_ inhibition by the drug was also attenuated when inward currents measured in high [K^+^]_e_ [47]. The dependence of I_hERG_ inhibition on [K^+^]_e_ was still evident for mutant channels with impaired inactivation, indicating that reduced I_hERG_ block by E-4031 with high [K^+^]_e_ was not secondary to reduced inactivation in high [K^+^]_e_ and was instead most likely attributable to electrostatic repulsion or ‘knock-off’, due to interactions between the drug and the permeant ion [47]. Our data on voltage- and time-dependence of inhibition and on WT I_hERG_ block in high [K^+^] (Figure 2, Figure 3 and Figure 5) are consistent with these earlier observations. The leftward shift in the voltage-dependence of WT I_hERG_ (Figure 4) inactivation is consistent with some stabilization of the inactivated state by E-4031, whilst the large reduction in block by the N588K and S620T mutations is consistent with a requirement for an intact inactivation process in order for high-affinity inhibition by E-4031 to occur [47]. Prior experiments on concatenated hERG channels have shown that replacement of a single serine at S620 with threonine in the tetramer disrupted inactivation completely, whilst potency changes for dofetilide and MK499 were graded depending on the number of S620T mutations in the tetramer [48]. Sensitivity of S620T to drug block was enhanced by a concomitant mutation Y652W, leading to a suggestion that this inactivation attenuating mutation may allosterically alter the position of Y652 such that it is less favourable for high-affinity drug binding [48]. It is possible that a similar explanation accounts for the effect of inactivation mutations on E-4031 inhibition of I_hERG_. The effects of mutations at the base of the pore helix and to S6 aromatic residues on E-4031 inhibition of I_hERG_ seen here are compatible with those reported previously for this drug [18] and other methanesulphonanilides [16,17,18]. This is the first study to establish S5 F557 to be a determinant of high-affinity channel block by E-4031 [19,20]. 

### 3.3. E-4031-17 Electrophysiology Data in Context

As for E-4031, the range of voltage-dependence of E-4031-17 inhibition (Figure 3) corresponded with the rising phase of the activation relation for WT I_hERG_, indicating contingency of block on channel gating. The rather modest negative shift in activation V_0.5_ with E-4031-17 is unlikely to constitute a major difference in effect from E-4031. The difference between the compounds in block development during successive applications of the long duration depolarisation in Figure 2 is noteworthy. There was a marked difference between initial levels of block for E-4031 (Figure 2Cii) and E-4031-17 (Figure 2Dii) on successive (first and second) applications of the protocol and between end-pulse block on second and fifth commands. Methanesulphonanilides are known to be ‘trapped’ in I_Kr_/hERG channels [18,49,50,51], and the results with the sustained depolarisation protocol for E-4031 here are very similar to those reported previously for dofetilide, which shows a low propensity to dissociate from the resting state [28]. Our findings for E-4031-17 are suggestive that it can dissociate from the channel between commands more readily than does E-4031, although block was still able to accumulate with repeated protocol application. The relatively weak inhibition of inward I_hERG_ in high [K^+^]_e_ may indicate that this analogue can be relatively easily displaced by electrostatic repulsion and it is feasible that this may correlate with a reduced propensity for trapping [52]. While detailed investigation of drug trapping was beyond the intended scope of this study, future work to investigate use-dependent inhibition of I_hERG_ by E-4031-17, with mutagenesis to evaluate trapping/untrapping [18,51,53], may be instructive. Unlike E-4031, E-4031-17 did not produce a negative shift in voltage-dependence of inactivation of WT I_hERG_ (and so did not stabilize the inactivated state), and yet, I_hERG_ block by the analogue was highly sensitive to inactivation mutants. This is consistent with the idea, discussed above for E-4031, that inactivation competency is important for the orientation of key binding residues rather than for binding to inactivated channels per se. The compound’s interaction with residues implicated in drug block of hERG is considered below.

### 3.4. Blocking Determinants for E-4031 and E-4031-17

As for E-4031, the range of voltage-dependence of E-4031-17 inhibition (Figure 3) corresponded with the rising phase of the activation. Kamiya et al. found that the T623A mutation reduced the inhibitory action of E-4031 on I_hERG_ carried by hERG expressed in Xenopus oocytes and suggested hydrogen bond formation between the side chain of T623 and the methanesulphonate group of E-4031 [18]. In contrast, Imai et al. were unable to find a direct interaction between T623 and E-4031 in a hERG model based on the bacterial K^+^ channel, MthK [54]. They suggested that T623 does not directly contact E-4031, but instead may interact with Y652 in order to stabilise conformations of the aromatic side chains optimal for drug interaction. Although in our experiments I_hERG_ block by E-4031 and E-4031-17 was reduced for T623A I_hERG_ compared to WT I_hERG_, the reduction was modest (particularly for E-4031-17). This may be more consistent with a supportive than direct binding role for T623, potentially through hydrogen bonding with Y652 side chains which promotes side chain conformers optimal for π-π stacking or cation-π interactions. The S624A mutation produced a notable reduction in I_hERG_ block by both compounds, indicative of an important role of this residue in drug action. We conducted exploratory docking simulations (not shown) employing both a cryo-EM structure [55] and a MthK-based homology model [56], using previously described methods [20]. E-4031 could not make simultaneous contact with S624 and aromatic residues in the cryo-EM model, but the sulphonamide group could form hydrogen bonds with S624 in the MthK-based model. As E-4031-17 lacks hydrogen bonding potential, rather than interacting directly with S624, it is more likely that the serine residues help to stabilise the protonated tertiary ammonium group of E-4031-17 in or near the binding site for the cavity K^+^ ion seen in potassium channel crystal structures [56,57]. The sensitivity of E-4031-17 to “knock off” with inward K^+^ flux is certainly consistent with a location of part of the molecule in the K^+^ ion conduction pathway.

When F557 was first identified as an I_hERG_ blocking determinant, docking using a modified hERG_KvAP_ template revealed high-affinity inhibitors including dofetilide are able to partition into lateral, hydrophobic pore openings [19]. In the cryo-EM open-inactivated structure, four hydrophobic pockets were identified below the selectivity filter that are lined by the well-known putative hERG binding residues [55]. The side chains of F557 residues can sit between Y652 and F656 residues on the same subunit and are, therefore, available to interact with drugs that enter the pocket [55]. In a recent Rosetta in silico modelling study [58], employing cryo-EM hERG structural data [55], no interactions between E-4031 and F557 were reported for wild-type hERG, although simulations of a fast inactivating mutant S641A resulted in lateral fenestrations and some configurations in which the drug’s pyridyl group could reach F557 [58]. It is noteworthy, therefore, that our experimental data showed marked effects of the F577L mutation on I_hERG_ block by both E-4031 and E-4031-17. Saxena et al. highlighted that F557L and Y652A produced comparable levels of attenuation of drug block of I_hERG_ [19] and this was also observed in subsequent studies [20,21,59], suggesting that the contributions of the side chains from these two residues are linked [19,60]. Consistent with this, in limited docking simulations using the cryo-EM structure (not shown), we observed that both E-4031 and E-4031-17 could interact with multiple Y652 side chains whilst making simultaneous interactions with an F557 aromatic side chain. 

Mutations at F656 have been shown to profoundly impair I_hERG_ inhibition by structurally and therapeutically diverse drugs (e.g., [4,16,17,18,20,22,29]). In homology models of hERG, F656 projects towards the K^+^ permeation pathway, enabling F656 residues from multiple subunits to interact with drugs [56,57,61,62]. Against this background, a surprising feature of the cryo-EM open channel hERG structure is that F656 projects away from the K^+^ conduction pathway and towards S5 [55]. A second published cryo-EM structure for hERG, with astemizole bound to the channel found that of nine hydrophobic interactions between the channel and drug, only one of these involved F656 [44]. In exploratory docking simulations using the cryo-EM structure, simultaneous interactions between both drug molecules and F656 and other binding residues only involved a single F656 residue, whilst with an MthK homology model, both molecules interacted with side chains of multiple F656 residues (not shown). These observations for E-4031 and E-4031-17 are in accord with prior findings for minimally structured high-affinity Cavalli-2 [20]. It is possible that interpretations of multiple side chain interactions with F656, based on homology modelling, are mistaken, and that the role of F656 is predominantly to stabilize the conformation of Y652 side chain for favourable drug interaction [20]. Experimental data from the use of tandem dimers to study high-affinity drugs including E-4031 suggest that > 1 F656 residue can be mutated without disturbing allosteric interactions important for high-affinity binding, but also indicate that if interactions with multiple F656 residues occurs, this must involve residues on opposite sides of the tetrameric pore [54]. However, a challenge to a primary role for F656 being to stabilize the conformation of Y652 for drug interactions is that some drugs (e.g., propafenone) are relatively resistant to mutation of Y652, but are highly sensitive to mutation of F656 [23]. One alternative possibility is that the extant cryo-EM structures have been captured in a conformation that is not optimal for high-affinity binding [44,55]. It is not possible to discriminate between these possibilities based on the data from this study; future work on this is likely to be valuable.

### 3.5. Conclusions

Removal of the methanesulphonate group of E-4031 does not adversely affect the hERG-blocking capability of the compound, nor does it eliminate a dependence of high potency inhibition on S5 and S6 aromatic residues. Polar substituents previously considered to be important determinants for drug interaction with amino acid side chains in the hERG pore are not necessarily required for high-affinity hERG block; the reduction of E-4031-17 inhibition by S624A suggests that this residue can influence block allosterically (likely through promoting interactions of the protonated nitrogen of the drug molecule with the cavity K^+^ binding site below the selectivity filter), as it lacks hydrogen bonding potential. The observed differences between E-4031 and E-4031-17 in accumulation of block during repeated application of sustained depolarisation voltage commands raise the possibility that E-4031-17 might exhibit altered use-/rate-dependence of action. Given the limitations of I_Kr_ blocking class III antiarrhythmics due to reverse rate dependence [63,64], future exploration of use-dependence of I_Kr_ inhibition and of the rate-dependent effects of E-4031-17 on ventricular repolarisation could be valuable.

## 4. Materials and Methods

### 4.1. hERG Expressing Cell Lines and Mutants Employed

Experiments on WT hERG current (I_hERG_) utilized a Human Embryonic Kidney (HEK 293) cell line stably expressing hERG, kindly donated by Prof Craig January [30]. The HEK 293 cell line stably expressing the S6 Y652A mutation was used as described previously [65]. Mutations at the base of the pore helix (T623A, S624A), S6 helix (F656V), and S5 (F557L) were studied using transient transfection of HEK 293 cells and were used as described previously [20,29,66]. The attenuated-inactivation mutants N588K and S620T were also studied through transient transfection, as previously [20,31,39].

### 4.2. Maintenance of Mammalian Cell Lines and Transfection

HEK 293 cells transiently or stably expressing wild-type (WT) or mutant hERG constructs were maintained as previously described [20,29]. Cells were maintained in 40 mm Petri dishes and incubated with 95% O_2_ and 5% CO_2_. Transient transfections utilized either Lipofectamine 2000^TM^ or Lipofectamine LTX (Invitrogen, Waltham, MA, USA), according to the manufacturer’s instructions. As described previously [20], the amount of hERG construct DNA transfected varied from 0.1 to 1 µg according to observed levels of functional expression. CD8 (0.1 µg) was used as a transfection marker, with successfully transfected cells identified using Dynabeads^®^ (Invitrogen). Cells were plated onto sterilized glass coverslip shards at least 24 h prior to electrophysiological recording [20].

### 4.3. Electrophysiological Recordings

Glass coverslip shards onto which cells were plated were placed into a recording chamber mounted on the stage of an inverted microscope (Nikon Diaphot, Nikon Instruments, Tokyo, Japan). Cells were superfused at 37 ± 1 °C with a Tyrode’s solution containing (in mM): NaCl 140, KCl 4, CaCl_2_ 2.5, MgCl_2_1, glucose 10, HEPES 5 (titrated to pH 7.4 with NaOH). The T623A mutation is poorly expressing and requires measurement of inward I_hERG_ in high external [K^+^] ([K^+^]_e_) [20]. High [K^+^] Tyrode for measurement of T623A I_hERG_ and its corresponding WT control contained 94 mM KCl and 50 mM NaCl, but was otherwise similar to the standard Tyrode’s solution. Pipettes for whole-cell patch-clamp recording (Schott number 8250 glass, A-M Systems Inc., Sequim, WA, USA) were pulled and polished (Narishige, PP 830 and Narishige, MF 83, respectively) to resistance values between 2 and 4 MΩ. The intracellular solution contained (in mM): KCl 130, MgCl 1, EGTA, MgATP 5, and HEPES 10 (titrated to pH 7.2 with KOH). The liquid junction potential (LJP) between the pipette solution and bath solution was calculated as 3.2 mV, and as this was small, it was not corrected. Series resistance was corrected by 70–80% [20]. E-4031 (Tocris, Bristol, UK) and E-4031-17 ([26] kindly provided by Prof A. IJzerman, Leiden University) were dissolved in DMSO to give stock solutions of 300 µM, 1 mM, and 10 mM. Aliquots of stock solution were added to Tyrode’s solution to give the final concentrations referred to in the Results section (final DMSO concentration was less than 0.1% for all experiments). During recordings, local superfusate was exchanged using a home-built heated solution exchange device that was capable of exchanging solution in < 1 s. I_hERG_ block by methanesulphonanilides is known to develop progressively [67]. Consistent with this, I_hERG_ inhibition developed slowly for E-4031 and E-4031-17; therefore, run-down correction was required for adjustment of fractional block monitored with the standard protocol (Figure 2; and [20]) used here for concentration-response data. WT I_hERG_ run down was monitored and calculated as 15.7 ± 3.7% (n = 7) after 10 min of recording. Run-down correction using the standard protocol in this way has been conducted previously (e.g., [20]). 

Electrophysiological recordings were made using an Axopatch 200B amplifier (Molecular Devices, San Jose, CA, USA) and a CV203BU head stage. A Digidata 1320 interface (Molecular Devices) was used for data acquisition. Data digitization rates used were between 10 and 25 kHz during all voltage protocols, and an appropriate bandwidth was set on the amplifier between 2 and 10 kHz [20]. Data were analysed using Clampfit 10.3 (Molecular Devices), Prism versions 4.03 and 5.03, and Excel 2013. Data are presented as the mean ±S.E.M. (Standard Error of the Mean) or as mean with ±95% CI (Confidence Intervals). Statistical comparisons were carried out using a two-tailed Student’s *t*-test (paired or un-paired as appropriate) and either one- or two-way analysis of variance (ANOVA) with a Bonferroni post hoc test. *p* values of less than 0.05 were considered statistically significant. Levels of significance are indicated on Figures and described in Figure legends. 

### 4.4. Equations Used for Analysis

Fractional inhibition of I_hERG_ was determined as follows:

(1) $$Fractional\;Inhibition=1-\left(\frac{I_{hERG-Drug}}{I_{hERG-Control}}\right)$$

for which I_hERG_-_Drug_ represents current amplitude in the presence of a given concentration of E-4031 or E-4031-17 and I_hERG-Control_ represents current amplitude in control solution prior to drug application.

Concentration-response relationships were generated by plotting the mean fractional inhibition of hERG tail currents evoked at −40 mV (protocol shown in Figure 2; fractional inhibition determined using Equation (1)) in the presence of different drug concentrations at steady state. Mean ±S.E.M. experimental points were fitted with a standard Hill equation:

(2) $$Fractional\;Inhibition=1/(1+\left(\frac{{IC}_{50}}{\left[drug\right]}\right)h)$$

where fractional inhibition at a given concentration of drug [drug] is calculated using Equation (1); the concentration of drug that produces half maximal inhibition is *IC*_50_ and the Hill slope is *h*.

The voltage-dependence of I_hERG_ activation was investigated by fitting normalised tail currents elicited using the protocol shown in Figure 2 with a Boltzmann equation as follows:

(3) $$I=I_{MAX}/(1+\exp\left(\frac{V_{0.5}-V_{m}}{k}\right))$$

In this equation, *I* is the amplitude of tail current following the test potential, V_m_, *I_MAX_* is the maximal current, *V*_0.5_ is the voltage that produces half-maximal activation, and *k* is the slope factor of the fitted relation.

The voltage-dependence of hERG inactivation was investigated by calculating I_hERG_ availability, using a three-step protocol (Figure 4A; also [20,29,65,68]). The amplitude of the resurgent currents elicited by the final step of the protocol was corrected for potential deactivation as described previously [39,69] and the elicited currents were normalised to the maximal current observed during this phase of the protocol. Normalised current was plotted against the value of the 2 ms test potential and fitted with a Boltzmann equation as follows:

(4) $$\frac{I}{I_{MAX}}=1-(1+\exp\left[\frac{V_{0.5}-V_{m}}{k}\right])$$

In this equation, *I* is the peak amplitude of the resurgent peak at the start of the third step, *I_MAX_* is the maximum current observed during the third step following the brief 2 ms step at the test potential (*V_m_*). *V*_0.5_ is the potential that produces half-maximal inactivation and *k* is the slope factor for the fitted relationship.

## Data Availability

All the research data are available in the article.
